# Symptom- and Prevention-Based Testing of COVID-19 in Nursing Home Residents: A Retrospective Cohort Study

**DOI:** 10.1177/23337214211055338

**Published:** 2021-11-11

**Authors:** Kelly C. Paap, Anouk M. van Loon, Sarian M. van Rijs, Esther Helmich, Bianca M. Buurman, Martin Smalbrugge, Cees M. P. M. Hertogh

**Affiliations:** 1 Department of Medicine for Older People, Amsterdam Public Health Research Institute, 1209 Amsterdam University Medical Center, Amsterdam, The Netherlands; 2 Amsta Healthcare Organisation, Amsterdam, The Netherlands; 3 Department of Internal Medicine, Section of Geriatric Medicine, Amsterdam Public Health Research Institute, 1209 Amsterdam University Medical Center, Amsterdam, The Netherlands

**Keywords:** long-term care facilities, SARS-CoV-2, cycle threshold value, oxygen saturation, older adults

## Abstract

Nursing homes (NH) residents with COVID-19 can either be tested because of presence of core symptoms (S-based) or because of transmission prevention (TP-based). The investigated study sample included all NH residents who underwent SARS-CoV-2 RT-PCR testing between March 16, 2020 and May 31, 2020 (*n* = 380). Clinical symptoms, temperature, and oxygen saturation were extracted from medical records, 7 days before to 14 days after testing. COVID-19 was confirmed in 81 (21%) residents; 36 (44%) S-based and 45 (56%) TP-based: 45. Cycle threshold (CT) values did not differ between the groups. In the 7 days prior to the test falling (32%), somnolence (25%) and fatigue (21%) occurred in both groups. Two days before the test, we observed a stronger decrease in oxygen saturation and an increase in temperature for the S-based group compared to the T-based group that remained up to 10 days after testing. Residents within the S-based group were 2.5 times more likely to increased mortality within 30 days than residents in the TP-based group (HR, 2.56; 95% 1.3–5.2). Although, 73% of the T-based group did eventually develop core symptoms. Thus, attention to falling and daily measures of temperature and oxygen saturation can contribute to earlier detection.

## Background

Residents in nursing homes (NH) account for a disproportionate number of COVID-19 related deaths, making up half of all COVID-19–related deaths in Western countries (Laboni et al., 2020). This disproportionately high number might be due to a fast and “invisible” transmission combined with the clinical frailty of the NH population who, in general, have severe medical comorbidities (Arons et al., 2020). The presence of chronic medical comorbidities and older age are associated with increased risk of mortality in the current pandemic (Novel, 2020). The high prevalence of functional and cognitive impairment contributes to the susceptibility and severe course of SARS-CoV-2 infections in NH residents (Rutten et al., 2020; Wang et al., 2020). Initially the case definition of COVID-19, and reason to do a Reverse Transcription Polymerase Chain Reaction (RT-PCR) test, was presence of at least one of the following core symptoms: fever of feverish feeling, cough, or dyspnea (Verenso, 2020). Yet, the first major study describing COVID-19 in a NH showed that more than half of all the positive tested patients (56.5%) did not have these core symptoms when tested positive, but other symptoms such as sore throat, chills, malaise, and diarrhea (Arons et al., 2020). From previous studies, it is known that residents in NH with infections frequently do not have fever (Rosenberg, 1987; Sloane et al., 2014; Yoshikawa & Norman, 1996). Most of the NH residents with COVID-19 also did not develop fever, but still had temperature elevations (Rudolph et al., 2020). If the focus for testing is only on residents with core symptoms, many residents with COVID-19 are missed, with consequences for transmission prevention. Therefore, testing based on transmission prevention is also required, despite the presence of symptoms.

Furthermore, self-reporting of complaints is often compromised in residents due to limited ability to communicate (e.g., in residents with dementia) (Huffman & Kunik, 2000). Not (being) able to recognize these complaints in time can contribute to the fast and “invisible” transmission (van den Besselaar et al., 2021). Therefore, although residents may only show a rise of temperature and not fever, measuring objective parameters like temperature and oxygen saturation may provide more reliable information in the NH population in addition to observing symptoms (Lam et al., 2016; Lansbury et al., 2017; Sayers et al., 2013).

This study, in which NH residents were tested either because of presence of core symptoms (symptom-based testing) or because of transmission prevention (transmission–prevention-based testing), aims to describe the clinical presentation and course of COVID-19 (including 30-day mortality) in both groups and differences between both groups. A second aim is to compare the cycle threshold (CT) value of the RT-PCR test between the two groups.

## Methods

### Study Population

This study was conducted in residents living in the NH Amsta (Amsterdam, The Netherlands), a 1185-bed skilled nursing facility with 18 locations spread over [Amsterdam].

### Study Design

In this retrospective cohort study, all residents who underwent a RT-PCR test from March 16, 2020 (the day we started testing), up to May 31, 2020 were included. RT-PCR tests were performed with a throat and nasopharyngeal swab. Patients who had a positive RT-PCR test were considered to be COVID-19 confirmed (CT value was also reported). The residents where the test was performed were divided into two groups: 1) The symptom-based test group (S-based), a test was performed when a resident had core symptoms according to the Dutch guidelines (fever (temperature ≥38.0°C/≥100.4°F), cough, or dyspnea) (Verenso, 2020). 2) The transmission prevention-based test group (TP-based), a test was conducted when a resident may have had contact with individuals with confirmed COVID-19 (both residents and health professionals), after hospitalization or outpatient visit, or when residents were newly admitted to the NH. Residents on some wards were tested several times because one of the residents turned out to be positive. It sometimes happened that residents with already confirmed COVID-19 were tested again on these wards.

### Data Collection

Data about age, gender, BMI, type of ward, comorbidities, and renal function of all residents that were tested on COVID-19 were extracted from the electronic health record (EHR) PinkRoccade Healthcare myCaress (myCaress). Data were pseudonymized and considered as part of the usual care data; therefore, no additional informed consent were requested.

For all residents with a confirmed COVID-19, we searched for clinical symptoms and for temperature and oxygen saturation data in the EHR from 7 days before testing up to 14 days after testing. The oxygen saturation was measured with a pulse oximeter and the body temperature was measured differently per ward, rectal or tympanic. The same measuring instrument was used for a resident in consecutive measurements. Both saturation and temperature were measured daily at a fixed time, mostly in the morning, as much as possible. If several measurements were registered, the first recorded measurement was used.

Clinical symptoms were extracted by reviewing the EHR, using registrations of physicians as well as of nursing staff. We searched for clinical symptoms that were new or significantly changed (i.e., a change from incidental to more frequent falling) for each individual resident. A symptom was regarded as new if it had not been reported in the previous 4 weeks, and changed if it significantly changed compared to the previous 4 weeks.

From the day that the first patient tested positive, it was agreed for the whole NH Amsta to measure the temperature and the oxygen saturation daily in all residents and to register it in the Electric Medical Record (EMR) (specific sections). Data about temperature and oxygen saturation were extracted from these specific sections. Data on 30-day mortality were collected from the EHR of individual residents, starting from the first day of the positive test.

### Statistical Analysis

Descriptive analysis was performed on patient demographic and symptom variables (temperature and oxygen saturation). To indicate the number of missing values, the N on which percentages were calculated are reported. All continuous data were presented as means with standard deviation and 95% confidence intervals (CI). To evaluate the differences in clinical symptoms, temperature, and oxygen saturation between the two groups of residents with confirmed COVID-19 (symptom-based testing vs. transmission prevention-based testing) group ANOVA’s were applied. We considered a *p*-value of <.05 to be statistically significant. Survival curves on 30-day mortality were estimated based on the days between the positive RT-PCR test and the date of death using Kaplan–Meier curves in residents with confirmed COVID-19 (symptom-based testing and transmission prevention-based testing). The mortality rate of residents with confirmed COVID-19 (symptom-based testing vs. transmission prevention-based testing) was compared using a Cox proportional hazard model, using three models. Model 1 unadjusted, model 2 included gender and age, model 3 included gender, age, and reduced renal function*.* Reduced renal function was significant in the univariate analysis and was much more common in the S-based group. Results are presented with 95% confidence intervals and all reported *p* values are two-sided.

Finally, using ANOVA, we compared the CT value of both groups (symptom-based testing vs. transmission prevention-based testing).

All analyses were performed with the use of the SPSS statistical package, version 26.0 (SPSS, Armonk, NY: IBM Corp).

## Results

**Figure 1.**
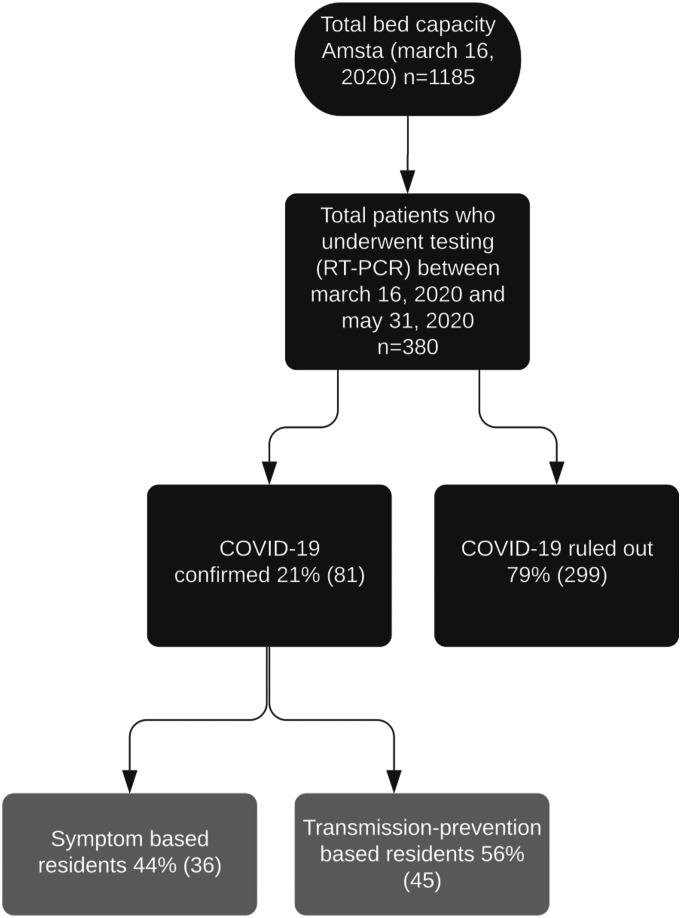
Flowchart.

On March 16, 2020, the date the first RT-PCR SARS-CoV-2 test took place, Amsta had a bed capacity for 1185 residents. Between March 16th and May 31st, 2020, 380 residents underwent a RT-PCR test for COVID-19 (601 tests). COVID-19 was ruled out in 299 (79%) residents and confirmed in 81 (21%) of residents. Of these 81 residents, 36 (44%) residents were tested because of presence of core symptoms (S-based), compared to 45 (56%) residents who tested positively but were tested because of transmission prevention (TP-based) (see Figure 1)

### Patient Characteristics

Mean age for the COVID-19 residents was 80 years (*SD* ± 11.2) in the S-based group and 82 years (*SD* ± 8.7) in the TP-based group. There were more women in both groups, S-based (53%) and TP-based (60%). In residents who were tested but for whom COVID-19 was ruled out the mean age was 79 years (*SD* ± 11.1), and the majority were woman 62%. Almost all residents in the TP-based group had dementia (96%), whereas in the S-based group this was 83%. In residents tested with COVID-19 ruled out, just over half of these residents had dementia (59%). After dementia, cardiac problems, hypertension, and a reduced kidney function were the most common comorbidities in residents who tested positive or negative for COVID. (Table 1.)

**Table 1.** Patient characteristics.

	Mean (*SD*), % (*n*)		
	Confirmed COVID-19 (symptom-based testing)	Confirmed COVID-19 (transmission prevention-based Testing	COVID-19 ruled out
N	44 (36)	56 (45)	79 (299)
Age, y	80 (11.2)	82 (8.7)	79 (11.1)
Sex			
Male	47 (17)	40 (18)	38 (115)
Female	53 (19)	60 (27)	62 (184)
BMI (kg/m^2)^	24 (4.6)	24 (5.1)	24 (5.3)
Ward type			
Psychogeriatric	58 (21)	91 (41)	46 (136)
Somatic	0	2 (1)	22 (67)
Short-term care	11 (4)	0	18 (53)
Geriatric psychiatry	6 (2)	0	9 (26)
Neuropsychiatry	25 (9)	7 (3)	6 (17)
Comorbidities			
Dementia	83 (30)	96 (43)	59 (175)
Cardiac history	31 (11)	33 (15)	42 (126)
Chronic respiratory disease	11 (4)	16 (7)	18 (55)
Hypertension	25 (9)	24 (11)	40 (120)
Renal function			
Normal renal function (eGFR ≥60)	28 (10)	73 (33)	51 (153)
Reduced renal function (eGFR <60)	64 (23)	9 (4)	40 (119)
No renal function registered	11 (4)	18 (8)	9 (27)

### Cycle Threshold Values

Cycle Threshold values were available for 64/81 positive residents; the mean CT value was 26.2 (range 12.5–40.4). Symptom-based tested residents (*n* = 27) had a median CT value of 24.8 (range 15.6–39), transmission prevention-based testing residents (*n* = 37) had a median CT value of 24.9 (range 12.5–40.4). There were no significant differences between these groups F (1.63) = .011, *p* = .919.

### Symptoms 7 Days Before Reverse Transcription Polymerase Chain Reaction-confirmed SARS-CoV-2 Infection

In the 7 days before a positively confirmed COVID-19 test, only fever was reported of the core symptoms and only in the S-based group. No core symptoms were present in the TP-based group. The most common other symptoms were: falling, somnolence, fatigue, and asthenia (Table 2). In the TP-based group of patients, falling occurred more often than in the S-based group (42% vs. 19%). Prior to the moment of testing other reported symptoms were: restlessness, dysphagia, diarrhea, sore throat, drowsiness, and headache.

**Table 2.** Core symptoms, other symptoms of COVID-19 confirmed residents.

	7 days before COVID-19 diagnosis		On Day RT-PCR		Up to 14 days after COVID-19	
	S-based COVID-19	TP-based COVID-19	S-based COVID-19	TP-based COVID-19	S-based COVID-19	TP-based COVID-19
Core symptoms % (*n*)						
Cough			75 (27)		42 (15)	13 (6)
Dyspnea			28 (10)		50 (18)	13 (6)
Fever	12 (4)		74 (25)		76 (36)	53 (24)
Other symptoms % (*n*)						
Agitated					6 (2)	31 (14)
Decreased appetite	6 (2)	11 (5)			64 (23)	38 (17)
Anxiety					28 (10)	9 (4)
Body weakness	11 (4)	27 (12)	19 (7)		47 (17)	24 (11)
Confusion		2 (1)			11 (4)	36 (16)
Diarrhea	28 (8)	4 (2)	19 (7)		17 (6)	24 (11)
Dysphagia	6 (2)	24 (11)	25 (9)	2 (1)	25 (9)	7 (3)
Falling	19 (7)	42 (19)				
Fatigue	17 (6)	24 (11)			17 (6)	27 (12)
Nausea					6 (2)	18 (8)
Restlessness	14 (5)	20 (9)				
Somnolence	17 (6)	31 (14)	33 (12)	2 (1)	31 (11)	13 (6)
Rhinorrhea	9 (7)		33 (12)			
Sore throat	3 (1)				3 (1)	
Drowsiness		4 (2)				
Headache	3 (1)		8 (3)			2 (1)

RT-PCR = reverse transcription polymerase chain reaction

### Signs/Symptoms on the Day of the Reverse Transcription Polymerase Chain Reaction-Test

On the day of COVID-19 testing, cough and fever were more frequent than dyspnea in the S-based group, 75%, 74%, and 28%, respectively. Other symptoms in the S-based group that were reported were somnolence (33%), rhinorrhea (33%), dysphagia (25%), body weakness (19%), and diarrhea (19%).

### Signs/Symptoms up to 14 Days After Reverse Transcription Polymerase Chain Reaction-Confirmed SARS-CoV-2 Infection

Fever is the most common symptom in the 14 days after the test, the majority of residents with a fever were in the S-based group (76% vs. 53% TP-based). The core symptoms cough and dyspnea were also most common in the S-based group compared to the TP-based group (cough 42% vs. 13%; dyspnea 50% vs. 13%). Of all residents with confirmed COVID-19 tested based on transmission prevention, 73% eventually developed core symptoms after an average of 4.2 days (*SD* ± 2.3).

Decreased appetite was almost twice as common in the S-based group as compared to the TP-based group (64% vs. 38%). Agitation and confusion were more often reported in the TP-based group compared to the S-based group (agitated 31% vs. 6% and confusion 36% vs. 11%). Body weakness and anxiety were reported more often in the S-based group (body weakness 47% vs. 24% and anxiety 28% vs. 9%). Furthermore, the following symptoms were reported: dysphagia and somnolence, both more often in the S-based group, 25% and 31%, respectively. Diarrhea, fatigue, and nausea were more frequently present in the TP-based group, 24%, 27%, and 18%, respectively.

### Day-to-Day Fluctuations in Temperature, 7 Days Prior and 14 Days After Testing

**Figure 2.**
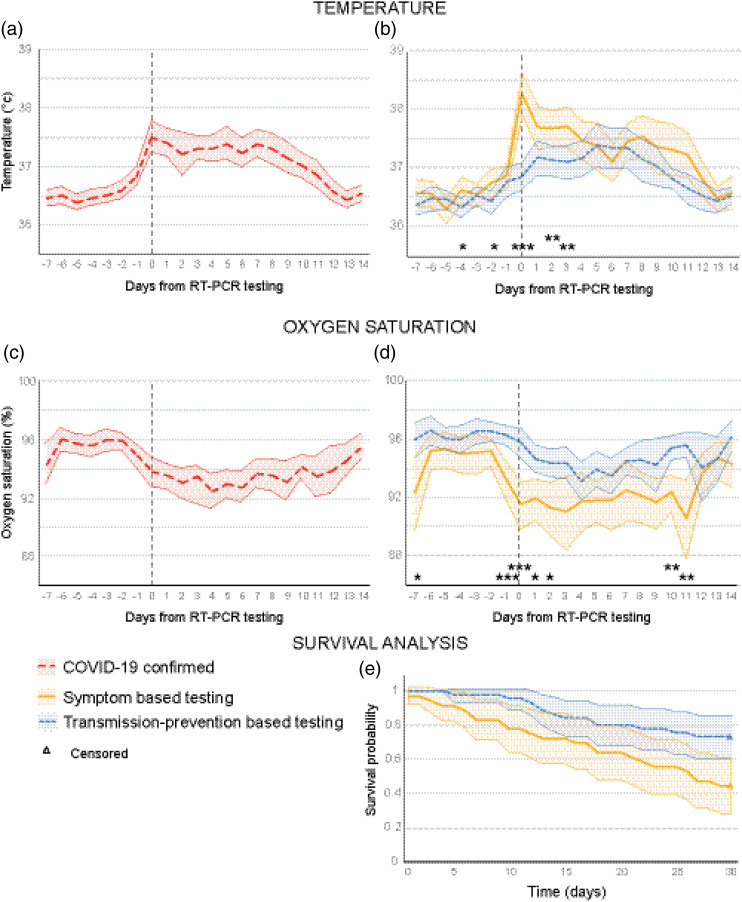
Temperature and oxygen saturation 7 days prior until 14 days after the Reverse Transcription Polymerase Chain Reaction test (T0) in NH residents with confirmed COVID-19 (*n* = 81). (a) Temperature for confirmed COVID-19. (b) Temperature split out for the symptom-based testing group (S-based *n* = 36) and the transmission prevention-based testing group (TP-based *n* = 45) (c) Oxygen saturation for confirmed COVID-19. (d) Oxygen saturation split out for the S-based and TP-based testing group. The shaded area represents the 95% confidence intervals. (*p < .05, ** *p* < .01, ***p < .001) (e). Kaplan–Meier estimates of survival for NH residents with COVID-19. NH = nursing homes.

The mean temperature of the residents with confirmed COVID-19 was lower in the 7 days prior to the positive RT-PCR test compared to the day of the RT-PCR test or up to ∼10 days after positive RT-PCR testing (Figure 2a). Up to two days before the positive confirmed COVID-19 test, an increase in temperature was observed, and on the day of the RT-PCR test the average temperature was highest. Seven days after the positive RT-PCR test, the temperature slowly decreased again. The temperature in the S-based group was higher before testing, on the day of the RT-PCR test and after the RT-PCR test compared to residents in the TP-based group (Figure 2b).

### Day-to Day Fluctuations in Oxygen Saturations, 7 Days Prior and 14 Days After Testing by Residents with COVID-19± and COVID-19+

The mean oxygen saturation by the COVID-19 confirmed group was higher in the 7 days prior to the positive RT-PCR test compared to the day of the RT-PCR test or up to ∼10 days after positive RT-PCR testing. The oxygen saturation dropped 2 days before the positive RT-PCR test. The oxygen saturation fluctuated until approximately 11 days after the positive confirmed COVID-19 test, after which it slowly increased again (Figure 2c). Overall oxygen saturation was lower in the S-based group prior to testing, on the day of the RT-PCR test and after testing in comparison to the TP-based group (Figure 2d).

### Mortality in Nursing Homes Residents with Confirmed COVID-19 in S-Based and TP-Based Testing Group

Of the residents with a confirmed COVID-19, 40% died within 30 days (95% confidence interval [CI], 29%–50%) More residents in the S-based group died 56% (95% CI, 40%–72%) compared to residents in the TP-based group 27% (95% CI, 14%–40%) (Figure 2e). Residents with in the S-based group were 2.5 times more likely to increased mortality within 30 days than residents in the TP-based group (adjusted hazard ratio (HR), 2.56; 95% 1.3 to 5.2; *p* = .010). This difference remained when adjusted for gender and age (HR of 2.4; 95% CI 1.2 to 4.9; *p* = .017) and when adjusted for gender, age, and reduced renal function (HR of 2.2; 95% CI 1.0 to 4.6; *p* = .038).

## Discussion

We are the first to show day-to-day fluctuations of the oxygen saturation in COVID-19 positive NH residents; oxygen saturation decreases approximately two days before testing in all residents who tested positive. We observed that oxygen saturation was lower for the S-based group prior to test than for the TP-based group. In addition, we observed an increase in temperature ∼3 days prior to test. Day-to-day fluctuations in temperature in residents with COVID-19 were described previously (Rudolph et al., 2020). Yet, our day-to-day temperature measurements add to this by making a distinction between NH residents with and without core symptoms on day of testing. We observed that temperature for the NH residents without core symptoms at day of test (TP-based) also increased 2 days prior to test. These results of the daily fluctuations in temperature and oxygen saturation (shifts 1–2 days before the positive test) can contribute to earlier testing and earlier detection of the “invisible” SARS-CoV-2 virus. If testing is done sooner, the residents can also be placed in a separated cohort earlier and the chance of possible spreading of the SARS-CoV-2 virus is smaller.

Furthermore, measuring the temperature and oxygen saturation on a daily basis is non-invasive and does not depend on the ability to express symptoms. Many NH residents frequently have difficulty putting their symptoms into words and therefore carry a high risk of not being recognized as COVID-19 patients. For example, our results demonstrated that almost all residents who were tested based on transmission prevention were living in a psychogeriatric ward and had dementia. By measuring daily changes in their temperature and oxygen saturation, and acting on this through testing and early quarantine, we may reduce the chance of transmission in this vulnerable group of NH residents. Of the residents with confirmed COVID-19, 44% of the residents were tested based on the presence of core symptoms (S-based) and 56% of the residents were tested based on transmission prevention (TP-based).

In the 7 days prior to the test, only fever was observed as core symptom in the S-based group. We found that falling and somnolence were the most common reported other symptoms prior to the test in the TP-based group. Thus, extra attention should be paid to these symptoms since they might indicate a possible infection. This is in line with previous studies, in which symptoms such as falling and delirium were reported, but in which debuts with core symptoms were more common (Guo et al., 2020; Niu et al., 2020; Olde et al., 2020). In the 14 days following the test, apart from the core symptoms, other symptoms that were common were decreased appetite, body weakness, dysphagia, somnolence, confusion, and being anxious. Residents with confirmed COVID-19 who were tested based on transmissions prevention (TP-based) and not because of the presence of core symptoms on day of testing did develop core symptoms (fever, cough, dyspnea) in 73% of the cases, but 27% did not. To prevent transmission, we cannot base the testing policy on solely testing residents with core symptoms. The results of this study ask for repeated testing of all residents that have been in close contact with a resident with confirmed COVID-19. Furthermore all residents living together with a resident with confirmed COVID should be isolated for a quarantine period and personal protective equipment should be used in the care of these residents. Repeated testing of all care personnel of these wards is also appropriate (van den Besselaar et al., 2021). Our results thus support the approach for repeated weekly testing until no more positive test emerges, irrespective of symptoms, in NH facilities that has been advocated since May 2020 (Gandhi et al., 2020; Verenso, 2020).

In our study, 40% of the residents with confirmed COVID-19 died within 30 days. This percentage is higher than the 34% reported by McMichael et al., (McMichael, 2020) and the 26% reported by Arons et al. (Arons et al., 2020). These differences may be due to a difference in the prevalence of dementia, in our study the prevalence was much higher compared to the other studies. The residents who have been admitted to the NH because of dementia are all in an advanced stages. Moreover, we observed that residents with core symptoms on day of testing (S-based testing) were 2.5 times more likely to increased mortality within 30 days than residents without these core symptoms on day of testing (TP-based). Thus, even though for transmission prevention all residents should be tested irrespective of symptoms, staff should still be more alert when the core symptoms are present since the mortality risk is higher for these residents as compared to residents without these symptoms. However, please note that mortality rate was still high 27% in the TP-based group and that a large part of the TP-based group did develop core symptoms after ∼4 days.

This study had some limitations that must be acknowledged when interpreting results. The study was carried out within one organization and based on EHR data. Because of the use of EHR data, we may have missed symptoms and also workload during the COVID-19 pandemic may have influenced symptom registration in the EHR negatively. As we used both care staff and physicians registration, this might have been somewhat overcome. Measuring the oxygen saturation and especially the temperature (tympanic and rectal) will not have been the same in every ward, but it is close to practice. A RT-PCR test has relatively low sensitivity (63–78%) (Zitek, 2020). Consequently, we will have missed cases of COVID-19.

## Conclusion

Many NH residents in this investigated sample with positive PCR did not have core symptoms when tested (fever, cough, and dyspnea) but had other signs/symptoms at the day of testing and in the week before the positive test. Yet, a large part of this group did develop these core symptoms after the testing day. Daily fluctuations in temperature and oxygen saturation can contribute to earlier detection. The results of this study underscore the importance of current testing policies that advice ample and repeated testing of all residents and personnel that are in close contact with a resident with confirmed COVID-19. This study shows that course profiles may be present. However, in order to substantiate this with more certainty, more research is needed that is prospective and longitudinal.
